# Development and Usability of the OHiFamily Mobile App to Enhance Accessibility to Maternal and Infant Information for Expectant Families in Ohio: Qualitative Study

**DOI:** 10.2196/53299

**Published:** 2024-11-08

**Authors:** Natalie Perme, Endia Reid, Macdonald Chinwenwa Eluagu, John Thompson, Courtney Hebert, Steven Gabbe, Christine Marie Swoboda

**Affiliations:** 1 Department of Biomedical Informatics The Ohio State University Wexner Medical Center Columbus, OH United States; 2 College of Medicine The Ohio State University Wexner Medical Center Columbus, OH United States; 3 The Center for the Advancement of Team Science, Analytics, and Systems Thinking in Health Services and Implementation Science Research (CATALYST) The Ohio State University Wexner Medical Center Columbus, OH United States

**Keywords:** health resources, pregnancy, patient engagement, mHealth, maternal, mobile health, app, focus group, landscape analysis, birth, preterm, premature, mortality, death, pediatric, infant, neonatal, design, development, obstetric, mobile phone

## Abstract

**Background:**

The Infant Mortality Research Partnership in Ohio is working to help pregnant individuals and families on Medicaid who are at risk for infant mortality and preterm birth. As part of this initiative, researchers at The Ohio State University worked to develop a patient-facing mobile app, OHiFamily, targeted toward, and created for, this population. To address the social determinants of health that can affect maternal and infant health, the app provides curated information on community resources, health care services, and educational materials in a format that is easily accessible and intended to facilitate contact between families and resources. The OHiFamily app includes 3 distinct features, that is, infant care logging (eg, feeding and diaper changes), curated educational resources, and a link to the curated Ohio resource database (CORD). This paper describes the development and assessment of the OHiFamily app as well as CORD.

**Objective:**

This study aimed to describe the development of the OHiFamily mobile app and CORD and the qualitative feedback received by the app’s intended audience.

**Methods:**

The researchers performed a landscape analysis and held focus groups to determine the resources and app features of interest to Ohio families on Medicaid.

**Results:**

Participants from several focus groups were interested in an app that could offer community resources with contact information, information about medical providers and information and ways to contact them, health tips, and information about pregnancy and infant development. Feedback was provided by 9 participants through 3 focus group sessions. Using this feedback, the team created a curated resource database and mobile app to help users locate and access resources, as well as access education materials and infant tracking features.

**Conclusions:**

OHiFamily offers a unique combination of features and access to local resources for families on Medicaid in Ohio not seen in other smartphone apps.

## Introduction

As of 2021, there were more than 350,000 health-related mobile apps (health apps) available in Apple and Android (Google) app stores, 90,000 of which were added in 2021 alone [1]. Overall health is influenced by a web of circumstances, including social determinants of health, which cover impacts of access to healthy foods, safe housing, comprehensive health care, and more. Facing difficulty in any one or more of these areas can negatively impact a person’s health and can be especially harmful for pregnant individuals, new mothers, and their children. There are mobile apps that help users locate resources during a crisis and others that curate local resources in various cities. To our knowledge, however, there are no mobile apps that comprehensively address these concerns for the pregnant and parenting population in Ohio.

Although nationally the infant mortality rate (IMR) has declined in recent years to 5.4 per 1000 live births in 2021, there are still relatively high infant mortality and preterm birth rates in Ohio [2]. There is also a racial disparity in many states, including Ohio, evident in the differing rates for non-Hispanic Black infants versus non-Hispanic White infants. In 2020, Ohio’s overall rate had improved from 6.9 per 1000 live births to 6.7 per 1000 live births in 2019 [3]. The non-Hispanic Black IMR was much higher at 13.6 per 1000 live births in 2020, compared with the non-Hispanic White IMR of 5.1 per 1000 live births in 2020, showing a persistent racial disparity in Ohio [3]. Previous research indicates that disparities in pregnancy outcomes and maternal morbidity are partially caused by environmental and social factors related to income inequality, including air quality; access to health care; food availability; access to public services; poor housing; and neighborhood factors such as crime, unemployment, and high poverty rates. Neighborhood factors, in particular, have been shown to disproportionately affect non-Hispanic Black mothers [4,5]. Addressing social and economic inequalities and improving access to health care has been linked to better pregnancy and birth outcomes and reduced racial health disparities among low-income individuals, highlighting the importance of addressing social determinants of health [6-8].

Working with the Ohio Departments of Medicaid, Higher Education, and Health, researchers at The Ohio State University and the Ohio Colleges of Medicine Government Resource Center are working together as the Infant Mortality Research Partnership (IMRP). As part of this initiative, a patient-facing mobile app, OHiFamily, was created. OHiFamily attempts to fill the gap in the mobile app market for at-risk pregnant individuals and families by providing detailed information on community resources in Ohio, allowing users to find local resources to meet their unique needs. The app also provides care tracking for breastfeeding and diaper changes, as well as educational resources on topics including safe sleep, prenatal visits, and baby development. The app was developed to help inform parents and caretakers, especially those most in need of information and support.

This paper outlines the development of OHiFamily, the development of the resource database within the app, and reports on initial usability studies with parents. The paper concludes with a comparison of OHiFamily with other mobile apps available for this population and future work.

## Methods

### Focus Groups

A convenience sample of participants was recruited through flyers and announcements at nonclinical community and social service organizations that focus on infant and maternal health, such as Moms2B group prenatal classes and women, infants, and children clinics [9]. Video conferencing sessions were set up through Microsoft Teams where feedback was requested. Participants and interviewers all participated remotely from their own homes or workplaces. Focus groups were led by a group of members of the research team (CMS, ER, and NP). The app developers did not attend the focus group but received feedback after the focus group responses were analyzed. All participants recruited were in varying stages of pregnancy and caregiving, including, mothers with recent births, currently pregnant individuals, mothers of young children, and 1 grandparent. The first 2 focus groups involved showing screenshots of the app as it was under development and asking participants their opinions on the features along with which community resources and assistance programs would be most helpful to them and their families. Participants viewed the app in action as focus group facilitators screen shared by a mobile device to show how to move through the app to access each feature. After the initial development of both Android and iOS (Apple Inc) versions of the mobile app, a third focus group was held several months later. Invited participants were able to download a demo version of the app to browse and view current available resources and features. Participants were given access to the mobile app a week before the focus group was held to give adequate time to review available features. The questions asked were the same for all 3 focus groups, the only difference being screenshots in the first 2 sessions and using the app live in the third session.

Participants were asked to complete a post–focus group survey through Qualtrics (Silver Lake), where they were also able to give additional feedback about the design and look of the app. This survey consisted of both multiple choice, free response, and Likert-type questions. Questions included in the focus group and survey asked whether the app looked easy to use, how often and when (during pregnancy or after) the app would be used, features they liked, and features that should be improved, potentially removed, or added. The survey also included a question asking the user to pick the 5 most important app features from a list. The focus group sessions were transcribed by a member of the research team, and thematic analysis was conducted by the research team to identify concepts that emerged. Preliminary codes were assigned to describe content and these codes were reviewed by the research team to determine themes. The analysis was complete when there were no additional codes that could be identified and saturation of responses was reached.

### Landscape Analysis and Creation of Curated Ohio Resource Database

Using feedback from the focus groups and from subject experts, a modified landscape analysis was conducted for community resources in Franklin County, Ohio. The data collected during this analysis laid the foundation for available Franklin County resources in the curated Ohio resource database (CORD) within OHiFamily. The landscape analysis used various domain types (ie, resource categories) generated by the research team, which were modified and updated over time. At the onset of the analysis, domains to be researched included categories such as housing, transportation, food, childcare, and baby or child supplies. As resources were collected over time, additional categories were added as needed and informed by feedback from the focus groups and research team.

During the landscape analysis, we used Google Advanced Search and preexisting databases including CAP4Kids (created by The Children’s Advocacy Project) to collect information on resources [10,11]. When using Google Advanced Search, “Franklin County, Ohio” was entered in the “all these words” text entry box for every search. The information entered in the “any of these words” text entry box varied based on the domain of interest and included several combinations of different keywords, such as “clinic” and “free clinic” for the health care domain. The first several pages of results were browsed, and keywords were altered if results were limited. Once the Google Advanced Search was completed, the team looked through corresponding categories in the CAP4Kids database and other informational websites such as Columbus Public Health [12]. The resulting organizations found in each domain were compiled into lists. The lists were analyzed, including visiting each organization’s website and browsing their available services, associated costs (if any), hours of operation, eligibility requirements, locations, and contact information. If the organization met our search criteria, the information for the resource was entered into a Microsoft Excel spreadsheet. If the inclusion of a resource was in question, the researcher consulted subject matter experts within the IMRP, including physicians and project managers, to determine the initial inclusion of the resource in CORD. Physicians on the project included those with expertise in obstetrics and gynecology, maternal-fetal medicine, pediatrics, infectious disease, and biomedical informatics.

**Figure 1:**
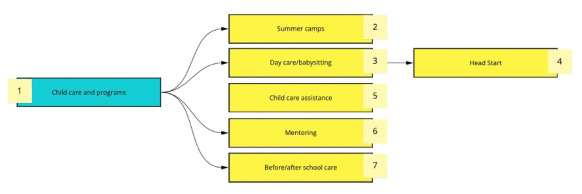
Branching logic of the curated Ohio resource database (CORD).

During the creation of CORD, we assembled a taxonomy to organize the resources by subcategory. Using a shared whiteboard from Miro (RealtimeBoard, Inc), the research team made a diagram to visually display the branching logic [13]. The branching logic and names of subcategories were altered over time to best reflect the resources within CORD and simplify filtering for the end user. A section of the taxonomy is shown in Figure 1.

Once the lists of resources had been made for each domain in Excel and the taxonomy was created, the data were manually transferred into JSON (JavaScript Object Notation) files to load directly into the web app, using the free JSON Formatter [14]. Members of Research Information and Technology (RIT) assisted the research team by creating templates for the resource files, which set up the logic for the strings, arrays, and objects of each resource entry. JSON files were uploaded to a cloud storage space accessible by the research team and select members of RIT. The creation of CORD has been an iterative process and resources have constantly been modified, removed, and added since starting this portion of the project in August 2020. If changes were made to a JSON file after the initial upload, a new version was uploaded into the cloud and RIT was informed of the change. At the time this paper was written, the mobile app and CORD were not yet publicly advertised; however, the publicly facing URL was shared with partner groups and stakeholders.

### Mobile App and Website Development

**Figure 2:**
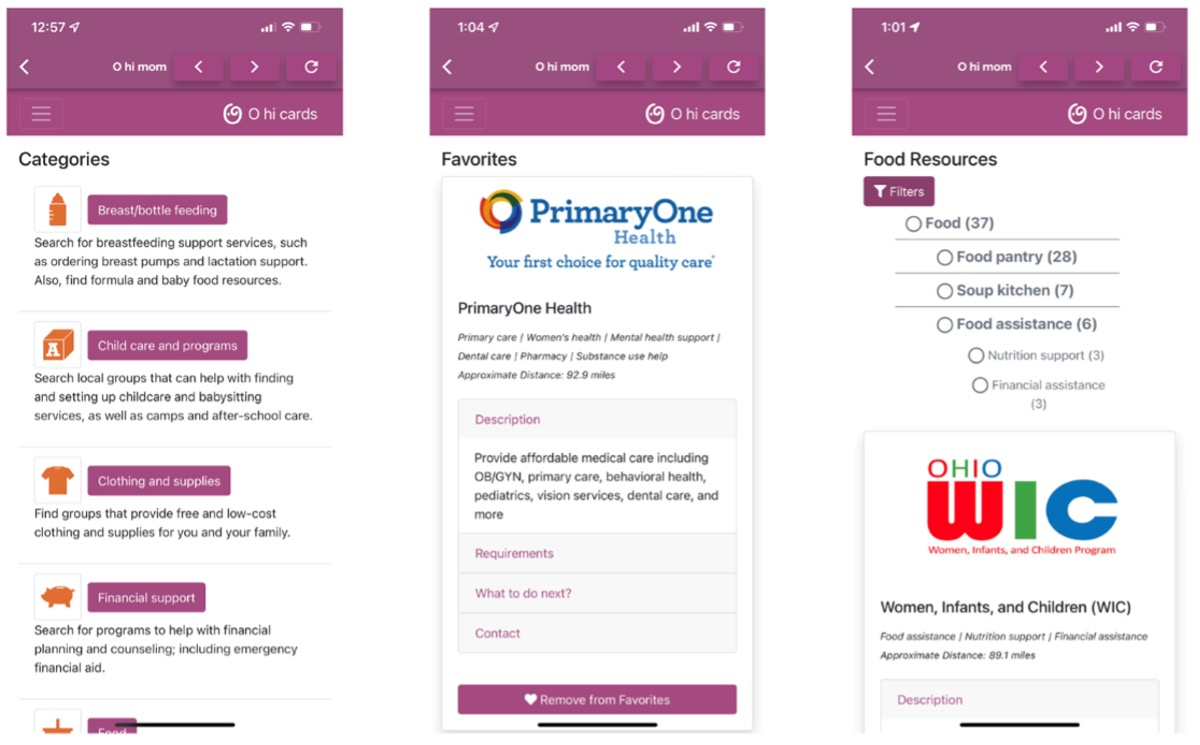
OHiFamily app resource section (ie, the curated Ohio resource database [CORD]).

**Figure 3:**
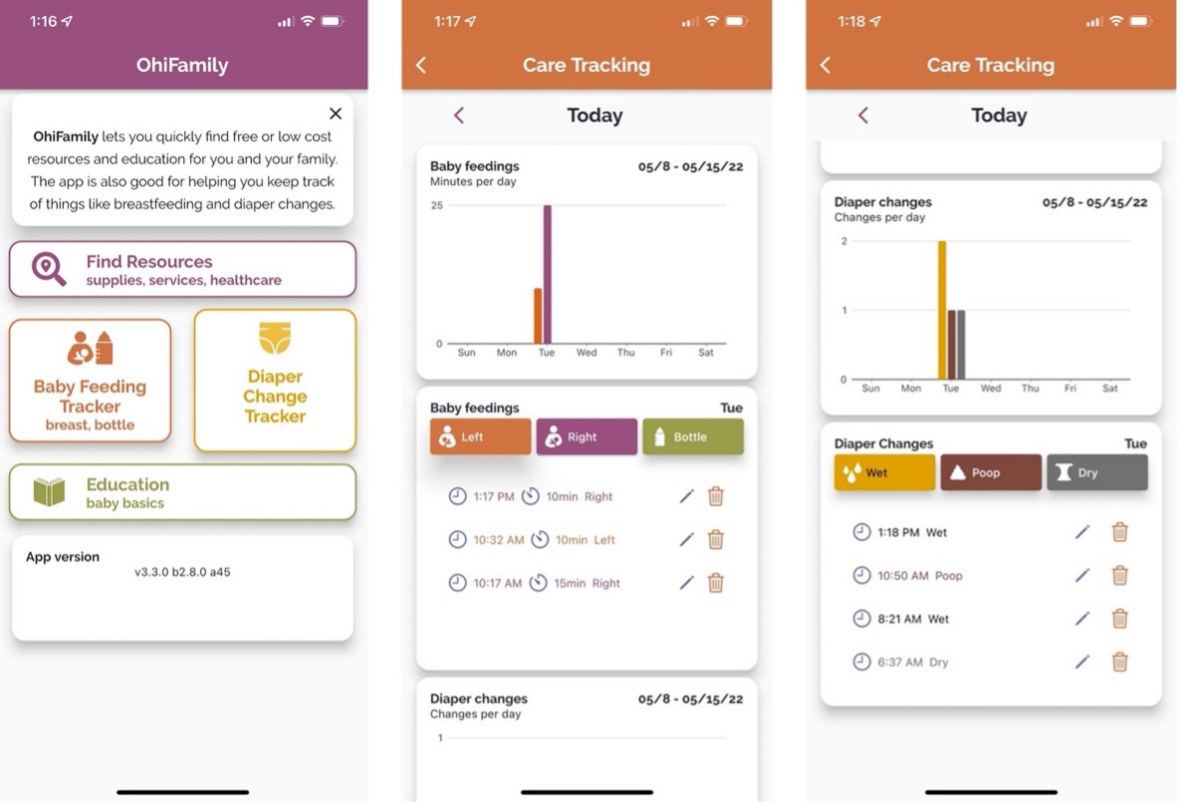
OHiFamily app home page and care tracking features.

RIT designed and developed the patient-facing mobile app guided by iterative feedback from the focus groups. Versions for both iOS and Android devices were created using Flutter (Google), an open-source framework that allows for multiplatform development from a single codebase [15]. The app provides tracking functionality (diaper tracker and breast- or bottle-feeding tracker) and allows for searching the CORD database. The app displays the CORD resources, stored in a MongoDB (MongoDB Inc) database using JSON files, as separate cards. Users can browse the cards as well as filter by subcategories corresponding to those present in the previously mentioned taxonomy. Screenshots from the resource section in early versions of the patient app are shown in Figure 2. Screenshots from the home page and tracking sections of the OHiFamily app (as of June 2022) are shown in Figure 3.

### Ethical Considerations

The referenced studies were approved by the Ohio State University Institutional Review Board (2020H0412), and informed consent was obtained from participants in all focus groups. Verbal informed consent was obtained before the focus groups by videoconferencing sessions on Microsoft Teams. All focus group data were deidentified before analysis. All focus group and survey participants were given US $20 Amazon gift cards for their participation. No identifying information is included in any images, and the app screenshots shown used a fake account created for research purposes.

## Results

### Results of Focus Groups

There were 3 focus groups, which included 2 participants in the first, 6 participants in the second, and 1 final participant in the last. The first 2 focus groups viewed demos of the app, while the third involved the user downloading the app to their personal phone before the session. Throughout the focus groups, the participants communicated that the app looked easy to use and not confusing but may be overwhelming to those who are not tech-savvy or those with language barriers. The app was designed for mothers of infants, but many participants expressed interest in using it throughout pregnancy and into early childhood. Participants liked the organized layout, the ability to directly contact resources, the availability of prenatal programs they might not otherwise have known about, and the relieved stress by having everything available in one place. Resources that participants wanted to see added included options for slightly older children like camps or mentoring, and culturally appropriate resources to support certain minority groups. Suggested app additions or improvements included the ability to track the weight and length of the child, keep track of appointments, a live call line to ask questions, ability to have multiple children in the app, connections to medical records, trackers for things like water and steps, nutrition suggestions, and information about the development of the child. Selected quotes from focus groups 1, 2, and 3 are presented in Table 1.

**Table 1:** Selected focus group themes and quotations regarding OHiFamily app feedback.

Theme	Selected quotes
Ease of use or barriers or difficulties using app	“I think it’s like any app, you can surf through it easily, the menu takes you where you need to get, I didn’t see any issues.” (focus group 1) “When you click on some things it can be redundant, both transportation and health help with transportation to doctor’s appointments, but it might be nice for people who are not as savvy.” (focus group 1) “Some moms could be slightly overwhelmed, when they look at it, it might seem like a lot to do, especially first time moms it might be overwhelming. If it’s explained, it might help.” (focus group 1) “Some people might not even speak the language, like is there an option to go through the app in a different language.” (focus group 1) “Yes, everything is right in front of you, you can click on each thing and its broken down easily, that’s one thing I liked that stood out for me.” (focus group 3)
When people would use this app	“Definitely the beginning and throughout the pregnancy, you go to so many appointments, you have a lot to keep track of. I had gestational diabetes, I had to keep track of my sugar. Moms have different things that happen when they’re pregnant. I would want to use it throughout my whole pregnancy and after.” (focus group 1) “Every day, just had a baby a month ago, this is like three of my apps all in one and I used at least one every single day during pregnancy.” (focus group 2) “Definitely could use it from first finding out about pregnancy to a year and a half or two years.” (focus group 2) “As long as a child has appointments you would continue to use it, sometimes you want to change places, different doctor offices, if you have to go to a specialty doctor for something in particular that would help with that, I feel like its something that you could reference on a consistent basis.” (focus group 3) “Right now, I would think its necessary up through the age of five, cause that’s when they start going to school” (focus group 3)
Features liked about app	“Everything looked amazing, it seemed like everything I would have loved as a pregnant woman, when I was trying to do stuff, they gave me all these books, and I was like okay, now what, you had to look through and do all these things...But if everything was at your fingertips...What if my reading level was at second grade but I know how to use an app? It seems like it could reach a lot of different people with a lot of different backgrounds.” (focus group 1) “I think they would feel they have the resources, sometimes people are worried about going to a pantry or a food bank, and if its explained how to do it, who to contact, it might give them hope and feel less stressed and anxious about everything.” (focus group 1) “I like how if I ask for transportation, it tells me exactly where I need to go, cause normally I would have to call like 5 people.” (focus group 1) “I like the organization, that everything has its place and its purpose, cause if I get four directions I’m gonna get lost, but if you give me the ability to touch a button and its organized, it helps do everything right.” (focus group 1) “I saw you had Moms2Be on there, I’m in Moms2Be, I would have never heard of it if it was not for another program that referred me, I think an app that refers to programs like that would be really beneficial” (focus group 2)
Resources to add	“Maybe more directions toward facilities that can do everything, like social work. Sometimes it’s easier for things to be a one stop shop, so an option for an agency or two. Definitely things like support groups, and the religious thing, we had a big group of people from India or Malaysia, and the people that had a center that deals with their particular community, with someone familiar that speaks their language.” (focus group 1) “I have four kids 9 and under, I was wondering if there will be summer camp programs, or learning programs, if there will be something like that for older kids?” (focus group 2) “Also reminders for school enrollment, like kindergarten or pre-K would be awesome too.” (focus group 2) “Is there a way to list churches where we can give back, cause I love to give back wherever I can, so like a list of who is accepting stuff, so if I can give diapers, would you be able to list something like that?” (focus group 2) “I think you covered all of em, housing, transportation, jobs, clothing...Food pantries...Finding doctors, that’s the biggest thing...” (focus group 3)
Features to improve or add	“Is there a live option? Like if I’m having breastfeeding issues at 2 o clock in the morning, is there a 24 hour nurse that could be accessible? Someone to call would be great. A live person and hooking up MyCharta would be an amazing addition. Is there a live option? Like if I’m having breastfeeding issues at 2 o clock in the morning, is there a 24 hour nurse that could be accessible? Someone to call would be great. A live person and hooking up MyChart would be an amazing addition.” (focus group 1) “Being able to toggle between multiple children, like if you have appointments for different kids, being able to separate those, having like an icon or profile for each one of your babies, for moms of twins or kids of different ages.” (focus group 2) “Will this be user friendly for men as well?” (focus group 2) “It would be good to be able to put in the medical information. When you go to appointments you get so much paperwork, being able to put that all in one app, keep track of growth, appointment things, I’d really like that” (focus group 2) “If you’re not using birth control or practicing safe sex, that could be something to add in, I would delete the one I have now.” (focus group 2) “I don’t know if it does tracking of your steps and stuff, I know a lot of apps have that, I don’t see it on here yet, so probably that. I know my Fitbit, I can put water in, so maybe amount of water a day, that’s something that is important.” (focus group 3) “I guess, things you can eat that will help with certain things, like I was anemic, my iron level was low, so it would like suggest eating this to help with that. Food suggestions, that would help.” (focus group 3) “I know I should be hearing kicks by now cause I’m this far along, am I supposed to be feeling this right now? That kinda helps, it’s this big, stuff like that.” (focus group 3)

^a^MyChart: name of the institution’s patient portal.

### Survey Results

Out of the 9 focus group participants, 7 completed the survey. Participants were given a checklist with 15 features and were encouraged to first check all that they would like to see, and then to select only their top 5. This list of 15 features was developed by a research scientist with experience developing mobile health apps. While multiple participants selected all of the features, there were some features that more often ranked near the top. The most selected features that participants wanted to see on a pregnancy app were information about community resources, information about medical providers, health tips, information about pregnancy and infant development, and phone numbers to call for medical providers and community resources. Results are shown in Table 2.

**Table 2:** Selected mobile app features selected as top priorities for pregnancy apps.

Feature	Responses selecting the feature in their top 5 (n=7), n (%)
Information about community resources (WIC^a^ and food stamps)	7 (100)
Information about medical providers	4 (57)
Other health tips (sleep, mental health, and medication or vitamin tracking)	4 (57)
Information about pregnancy or infant development	3 (43)
Phone numbers to call for medical providers or community resources	3 (43)
Phone numbers to call to talk to someone about your pregnancy concerns	2 (29)
Transportation resources to get help getting to appointments for free or cheap	2 (29)
Goal setting	2 (29)
Planning or calendar with reminders	2 (29)
Financial plannings or budgeting and price information	2 (29)
Shopping list for baby supplies needed or suggestions for baby supplies	1 (14)
Note taking area	1 (14)
Maps of where to find providers and resources	1 (14)
Healthy eating or exercise tips	0 (0)
Motivational videos or audio clips about motherhood	0 (0)

^a^WIC: women, infants, and children.

### CORD Refinement Based on Feedback

When CORD was constructed in early 2020, there were 14 categories and 89 subcategories. The major categories include breast- or bottle-feeding, childcare and programs, clothing and supplies, financial support, food, for dads, health care, housing, legal aid, pregnancy and parenting support, technology, training, jobs, and education, transportation, and trauma support. Within each of the categories are several subcategories that further filter the resources based on service type. The major categories now encompass 93 subcategories that have been updated over time. Resources for Franklin County were completed in April 2021. Resources for Hamilton and Montgomery Counties were compiled by the research team starting in late 2021 and finished in early 2022. Resources for Cuyahoga County and Summit County were curated starting in May 2022. As this is an iterative process, resources from these counties will be continually reviewed and updated as needed over time; however, the timeline of initial completion is mentioned here for reference.

## Discussion

### Principal Findings

This paper described the development of a patient-facing app (OHiFamily) and a geographically referenced resource database (CORD), as well as reporting of our findings from 3 focus groups for early versions of the app. Mothers in our focus groups were interested in tools that could help them with tracking infant care and identifying nearby resources. Based on feedback, our team was able to prioritize must-have features in the OHiFamily app, followed by additional “nice-to-have” features received positively by participants. Requests for resources targeting older children and specific cultural or ethnic groups were taken into account and new resources matching these descriptions were added to CORD where possible. To our knowledge, this is the only state-specific mobile app targeted at mothers to connect them with community resources.

Harnessing mobile health technology can enable a more widespread impact within a community. A recent survey from the Pew Research Center found that 85% of Americans own smartphones [16]. Pregnant individuals and parents are especially primed to use mobile technology, as there are more pregnancy-related apps than any other type of health app [17]. Data suggest that contemporary parents turn to mobile apps for many functions including information, education, and support on parenting topics ranging from safe sleep to breastfeeding [18].

When comparing OHiFamily with other mobile apps that assist with resource access, there are clear and important differences that make OHiFamily unique. The first app comparison was with SW Helper, created by progressive news group Social Work Helper. SW Helper is advertised as being able to help users locate resources during a crisis [19]. Categories include general resources such as food bank locator and highly specific categories such as Goodwill locator. Clicking on any category takes the user to an external search engine for that service type, where the user is then prompted to enter their zip code so nearby resources will populate. For example, clicking on the sexual assault center locator takes you to the RAINN website [20]. This differs from OHiFamily, where clicking into a category on the app allows you to browse through dozens of individual local resources in that category, as well as access external search engines for further browsing through these resource cards (eg, Action for Children is listed as a resource in the childcare and programs category but also operates its own childcare search engine). In addition, the SW Helper app is not specific to Franklin County or Ohio; the user must click into each external search engine and input their zip code to see resources in their area.

Another app, ShelterApp (Shelter App, Inc), allows user to find housing and other resources in their area including food, shelter, health, material resources, and work [21]. After allowing the app to access the user’s location, resources are sorted by distance with the closest resources populating first in each category. Clicking on any resource takes the user to a new page where the organization’s contact information, location, schedule, social media links, and websites populate. There is also a tab that displays crisis lines with brief descriptions of their services. This app differs from OHiFamily by having a broader focus, nonspecific to Franklin County or Ohio. This results in missing lesser-known but still important resources, such as certain church food pantries and free clinics with minimal advertising. Furthermore, by only offering 5 resource categories, the resources displayed do not give a comprehensive picture of the variety, and often needed services available to local families, especially those related to pregnancy and parenting.

The final 2 mobile apps used for comparison, 211 Central Ohio and Franklin County 211, are resource apps specific to the Central Ohio region and reflect a wide array of services in this area [22,23]. Although these apps are potentially more relevant to users in Ohio compared with SW Helper and ShelterApp, as they are curated with local resources ranging from mental health and counseling resources to housing and utility assistance. The target audience, however, for OHiFamily at its core is the Ohio Medicaid population of pregnant individuals, new parents, and families in Ohio who are at risk for preterm birth and infant mortality, which makes the app unique. As such, a large part of CORD is dedicated toward resources focused on new parents and families including breast- or bottle-feeding, childcare and programs, pregnancy and parenting support, and programs for fathers. When examining similar categories in the compared apps (such as doula services and pregnancy counseling), the research team noticed many organizations missing in Franklin County that would be useful for families to know about as they provide important services.

A recent focus group study on mobile health apps found that while over half the participants had downloaded mobile health apps previously, there were many barriers to initial download and continued use [24]. Participants without mobile health apps stated they experienced barriers due to low awareness of the mobile apps, lack of need for the mobile apps, lack of app literacy, and potential cost issues. Other data suggest that credibility of an app is one of the most important components for parents, and users are more likely to use an app recommended by a health care provider or friend [1]. Engaging primary care and obstetrics and gynecology providers to recommend and disseminate OHiFamily will help overcome many of these initial barriers. Recommendations from a trusted member of the medical community might also spread awareness and show patients credibility of the app, encouraging initial downloads. In addition, partnering with various community-based organizations will allow the recommendation of OHiFamily to their clients as well, serving as additional trusted sources and providing further credibility. With future updates to the app including the ability for users to comment and create or add to the resource list, parents will also be able to see reviews from peers and further judge which resources might be useful to them. Potential issues of app literacy, or not knowing which or how to use health and wellness apps could also be addressed through a provider, peer recommendation, or training. Finally, OHiFamily is cost-free with no in-app purchases, making it an attractive resource for parents trying to cut costs. A recent study among peripartum individuals found that more than half of their participants reported general financial stress, thus a free app that also provides various free and low-cost resources would likely garner interest [25].

Data suggest that time and effort required by many health and wellness apps and a lack of motivation prevent users from continuing to use them in the long term [24]. OHiFamily will provide continued usefulness through access to CORD; a curated resource database accessible at any time would not be expected to decrease in value over time, as resources within CORD will be continually added and updated. Being able to tailor resources for specific needs will also allow parents to use the app for different purposes as their children age; users can find items like diapers when their child is young and programs like summer camps when their child is older. In addition, parents will have continued access to many different resources for themselves such as foreclosure prevention counseling, legal assistance, and job training. The app requires only as much time and effort as the user is willing to give. If the user wants to access one kind of resource, they can quickly search and filter the resources to find what they need; they can also scroll through many categories of resources or use features like the breastfeeding and diaper trackers if they are useful to them.

Having resources for fathers alongside the resources for mothers is an especially unique and important feature of OHiFamily. While there are thousands of pregnancies and parenting apps, most of them separately target mothers or fathers. Our app provides features relevant to both groups, with a plethora of resources for moms, dads, caretakers, and families all in one place. In addition, the tracking features can be used by either parent, or another caregiver, to keep track of feeding and diaper changes. Feeding and diaper trackers were initially presented to focus group members, which they liked. They expressed the desire to have appointment and growth trackers in addition to the infant feeding and diaper trackers.

There are some limitations to our work. First, there was difficulty recruiting parents for our focus groups. This was partially because the app was tested during the COVID-19 pandemic, resulting in challenges recruiting in person at community-based organizations. Participants were only interviewed over videoconferencing, meaning that participants who already had internet access and time to meet were only included, limiting our generalizability. In addition, participants were presented with an early version of the app during the initial focus groups. Since these meetings, we have completed a heuristic review with our user experience team and plan to make several changes that will affect the user experience of the app.

### Future Work

The next step in evaluating this app is to provide the app to expectant mothers as part of a longitudinal study. This is currently ongoing. If funding supports continued updates, users will be able to create profiles for themselves and their children, a way to personalize the app to fit their family’s needs. Parents and caretakers will be able to link accounts and keep track of their children’s shared tracking information on their individual devices. Having the tracking features, CORD, and educational materials all in one place makes OHiFamily unique and potentially valuable for local families. In addition, the features available within the app have been tailored such that users can access the information in both English and Spanish. This was not a feature found in the 4 resource apps of comparison. Based on feedback from previous focus groups, it is understood that accessibility for families speaking non-English languages is valuable, and the team plans to add more languages in the future to reflect Ohio’s diverse immigrant population [26]. If possible, the team hopes to use information from user profiles to analyze which subsets and groups of community members are using the app the most, looking at various social determinants of health. In addition, the team is planning formal usability studies on the app as it continues to be updated and expanded to ensure the best experience for users.

### Conclusions

In this paper, we described the development and initial user testing of a patient-facing app, OHiFamily, intended to help parents access community resources. There has been generally positive feedback from parents about the app and our goal is to continue to add to its functionality based on the feedback. The app is unique compared with currently available community resource–focused health apps, in that it is specific to Ohio and to parents with infants and young children. Disparities continue to persist in infant mortality and severe maternal morbidity. Community resources can help address modifiable risk factors and social determinants of health by making it easier for parents to find smoking cessation programs, free medical clinics, safe housing, pregnancy support, and more. The OHiFamily app helps parents access these resources more easily.

## Abbreviations

CORD: curated Ohio resource database
IMR: infant mortality rate
IMRP: Infant Mortality Research Partnership
JSON: JavaScript Object Notation
RIT: Research Information and Technology

## Data Availability

The curated Ohio resource database (CORD) is publicly available at OHiFamily website and will be up through June 2025. The other data generated during and/or analyzed during this study are available from the corresponding author on reasonable request.
